# Diagnostic performance of thyroid ultrasound in Hürthle cell carcinomas

**DOI:** 10.20945/2359-3997000000131

**Published:** 2019-04-15

**Authors:** Nathalie Oliveira Santana, Ricardo Miguel Costa Freitas, Vinicius Neves Marcos, Maria Cristina Chammas, Rosalinda Yossie Asato Camargo, Cláudia Kliemann Schmerling, Felipe Augusto Brasileiro Vanderlei, Ana Oliveira Hoff, Suemi Marui, Debora Lucia Seguro Danilovic

**Affiliations:** 1 Universidade de São Paulo Hospital das Clínicas Faculdade de Medicina Universidade de São Paulo São Paulo SP Brasil Laboratório de Endocrinologia Celular e Molecular (LIM25), Hospital das Clínicas da Faculdade de Medicina, Universidade de São Paulo, São Paulo, SP, Brasil; 2 Universidade de São Paulo Instituto do Câncer do Estado de São Paulo Faculdade de Medicina Universidade de São Paulo São Paulo SP Brasil Radiologia, Instituto do Câncer do Estado de São Paulo, Faculdade de Medicina, Universidade de São Paulo, São Paulo, SP, Brasil; 3 Universidade de São Paulo Hospital das Clínicas Faculdade de Medicina Universidade de São Paulo São Paulo SP Brasil Radiologia, Instituto de Radiologia (InRad), Hospital das Clínicas da Faculdade de Medicina, Universidade de São Paulo, São Paulo, SP, Brasil; 4 Universidade de São Paulo Instituto do Câncer do Estado de São Paulo Faculdade de Medicina Universidade de São Paulo São Paulo SP Brasil Departamento de Patologia, Instituto do Câncer do Estado de São Paulo, Faculdade de Medicina, Universidade de São Paulo, São Paulo, SP, Brasil; 5 Universidade de São Paulo Hospital das Clínicas Faculdade de Medicina Universidade de São Paulo São Paulo SP Brasil Departamento de Cirurgia de Cabeça e Pescoço, Hospital das Clínicas da Faculdade de Medicina, Universidade de São Paulo, São Paulo, SP, Brasil; 6 Universidade de São Paulo Instituto do Câncer do Estado de São Paulo Faculdade de Medicina Universidade de São Paulo São Paulo SP Brasil Departamento de Endocrinologia, Instituto do Câncer do Estado de São Paulo, Faculdade de Medicina, Universidade de São Paulo, São Paulo, SP, Brasil

**Keywords:** Ultrasound, thyroid cancer, Hürthle cell, Doppler

## Abstract

**Objective:**

Hürthle cell carcinomas (HCCs) of the thyroid have been recently reclassified as a separate entity due to their distinct clinical and molecular profiles. Few studies have assessed the ability of preoperative characteristics in differentiating HCCs from Hürthle cell adenomas (HCAs) due to the low prevalence of both lesions. This study aimed to compare the preoperative features of HCCs and HCAs and evaluate the diagnostic performance of ultrasound in distinguishing between both.

**Subjetcs and methods:**

Retrospective study including 101 patients (52 HCCs and 49 HCAs) who underwent thyroid surgery from 2000 to 2016. Clinical, ultrasonographic, and histological data were reviewed. Diagnostic performance of suspicious sonographic features was analyzed in 51 cases (24 HCCs and 27 HCAs).

**Results:**

Hürthle cell neoplasms were predominant in females. Subjects ≥ 55 years represented 58% of the cases of HCCs and 53% of those of HCAs. Carcinomas were significantly larger (p < 0.001), and a tumor size ≥ 4 cm significantly increased the risk of malignancy (odds ratio 3.67). Other clinical, cytologic, and sonographic data were similar between HCCs and HCAs. Among the HCCs, the lesions were purely solid in 54.2%, hypoechoic in 37.5%, and had coarse calcifications in 12.5%, microcalcifications in 8.3%, irregular contours in 4.2%, and a taller-than-wide shape in 16.7%. Predominantly/exclusive intranodular vascularization was observed in 52.6%. Overall, 58% of the HCCs were classified as TI-RADS 4 or 5 compared with 48% of the HCAs. TI-RADS 4 or 5 had a specificity of only 51.8% and a positive likelihood ratio of 1.21.

**Conclusions:**

Apart from the lesion size, no other preoperative feature adequately distinguished HCCs from HCAs. Sonographic characteristics raising suspicion for malignancy, which are mostly present in papillary carcinomas, were infrequent in HCCs. New tools must be developed to improve preoperative diagnosis and deferral of surgery in cases of adenomas.

## INTRODUCTION

Thyroid carcinomas have increased in prevalence over the last decades (1). Papillary and follicular thyroid cancers are the most common histological types of thyroid cancer, while Hürthle cell carcinomas (HCCs) correspond to only 3 to 4% of all thyroid malignancies. Due to their distinct clinical behavior, HCCs have been recently reclassified as a separate entity (2,3).

A thyroid tumor is classified as a Hürthle cell neoplasm (HCN) when over 75% of its tumor cells display oncocytic histologic features without nuclear characteristics of papillary carcinoma. These so-called Hürthle cells are found not only in HCN, but also in benign lesions like nodular goiter, Graves’ disease, and Hashimoto’s thyroiditis, as well as after cervical radiotherapy. Oncocytic changes were previously considered to be a consequence of cellular senescence, but are now seen as a metaplastic process in response to a variety of stimuli inducing cellular stress (4-6).

Similar to follicular neoplasms, HCCs are distinguished from Hürthle cell adenomas (HCAs) by the presence on histological assessment of vascular and capsular invasion or identification of nodal and/or distant metastases (4). HCCs are classified as minimally or widely invasive, the last one presenting a poorer response to radioactive iodine therapy and higher recurrence and mortality rates (7-11). Fine-needle aspiration biopsy (FNA) of HCNs are usually indeterminate for malignancy, challenging the preoperative diagnosis of HCCs. Even current molecular preoperative tests are unable to distinguish HCNs and HCCs correctly (12). Therefore, surgery is still required to distinguish benign and malignant HCNs (13-15).

Only a few studies have reported the imaging findings of Hürthle cell tumors. Besides, due to their rarity, HCCs have been poorly represented in these studies, and their sonographic features have been analyzed in combination with those of adenomas (16-21).

The aims of this study were to compare the clinical presentation of HCCs and HCAs and evaluate the impact of thyroid ultrasound in distinguishing one lesion from another.

## SUBJECTS AND METHODS

### Subjects

This retrospective study included 101 patients with pathologically proven HCNs who underwent total thyroidectomy or lobectomy in a tertiary center from 2000 to 2016. Clinical, cytologic, and histopathologic data were collected by retrospective chart review. The histopathology of the lesions was reassessed by a pathologist.

The study was approved by the Research Ethics Committee of the University of São Paulo. Data were analyzed anonymously, and any identifying marks in the ultrasound images were removed.

### Methods

Preoperative ultrasound images were available in 51 cases (24 HCCs and 27 HCAs). Gray-scale and color Doppler images were obtained using high-frequency transducers (7.5-12 MHz) with the following US equipment models: iU22 (Philips, Eindhoven, The Netherlands), MyLab 70XV (Esaote, Florence, Italy), Sonix (Ultrasonix, Burnaby, Canada), Aplio 500 (Toshiba Medical Systems, Tokyo, Japan), or Logic E9 (GE Healthcare, Milwaukee, Wisconsin, USA). Two blinded radiologists retrospectively analyzed the sonographic imaging characteristics and, when their diagnoses were discordant, a third radiologist reviewed the images. The following nodular features were evaluated: size, echogenicity, solid and/or cystic aspect, contours, presence of a hypoechoic peripheral halo (rim) or calcifications, vascularization pattern, and presence of atypical lymph nodes. We compared the longest diameter of the lesions, and evaluated if the nodular height was greater than the width. The echogenicity of the nodule was assessed in relation to the normal thyroid parenchyma. The margins of the nodules were classified as regular or irregular. Microcalcifications were defined as hyperechoic foci up to 2 mm in size, while coarse calcifications were defined as hyperechoic foci > 2 mm in size with posterior acoustic shadowing. The type of vascularization was classified as peripheral, if the vascularization was predominantly perinodular, or central, if it was predominantly intranodular.

We analyzed the diagnostic performance of the following features of malignancy suspicion in HCCs: hypoechogenicity, solid aspect, irregular contours, absence of a hypoechoic peripheral halo, presence of microcalcifications or coarse calcifications, taller-than-wide shape, and intranodular vascularization pattern.

The nodules were classified according to the Thyroid Imaging, Reporting and Data System (TI-RADS) (22). The diagnostic performance of HCCs in the TI-RADS 4 and 5 categories, which correspond to moderately and highly suspicious nodules, respectively, was also evaluated.

### Statistical analysis

The data were processed using IBM SPSS Statistics for Windows, Version 24.0 (IBM Corp., Armonk, New York, USA). Two-tailed *p* values were used, and values < 0.05 were considered statistically significant.

Categorical variables are presented as absolute and relative (percentages) frequencies. Differences were evaluated by Pearson’s chi-square test and Fisher’s exact test, when appropriate. Continuous variables are presented as mean ± standard deviation values. Differences among study subgroups were determined using Student’s *t* test, for normal distributions, or Mann-Whitney U test for non-normal distributions.

The diagnostic performance (sensitivity, specificity, positive likelihood ratio [LR], negative LR) of the thyroid ultrasound exams was analyzed. We defined the true positive, true negative, false positive, and false negative results based on the final histological diagnosis (carcinoma or adenoma).

## RESULTS AND DISCUSSION

Data from 52 HCCs and 49 HCAs were available for review. No significant clinical differences were observed between benign and malignant cases. HCNs predominated in female subjects (83% HCCs vs. 84% HCAs). The mean age of the patients was 57.2 ± 14.5 years in malignant and 55.2 ± 13.3 years in benign cases. Subjects aged ≥ 55 years comprised 58% of the patients with HCC and 53% of those with HCA. The mean preoperative TSH level was 1.9 ± 1.5 µU/mL in patients with HCC and 2.1 ± 1.2 µU/mL in those with HCA.

**Figure 1:**
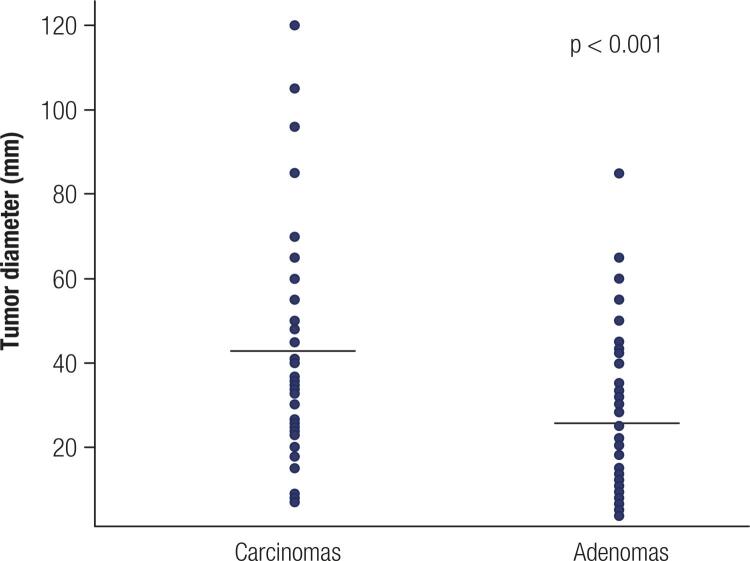
Comparison of the largest diameters of Hürthle cell carcinomas and adenomas. The solid lines represent the median diameter of the carcinomas and adenomas.

On pathological analysis, the diameter of the carcinomas was significantly larger (mean 47.4 ± 25.7 mm, median 46.5 mm) than that of the adenomas (mean 28.6 ± 19.5 mm, median 25 mm; p < 0.001). Nodules ≥ 4 cm had an increased risk of malignancy (odds ratio 3.67, 95% confidence interval 1.58 – 8.52) (Figure 1). Multinodular goiter coexisted in 51% of the carcinomas and 40.8% of the adenomas, while lymphocytic thyroiditis was found in 15.7% and 14.3% of the carcinomas and adenomas, respectively. Coexisting papillary thyroid carcinoma was diagnosed in 16% of the subjects with HCC and 20.4% of those with HCA. One patient with HCC had a concomitant poorly differentiated thyroid carcinoma.

All HCCs presented as unifocal lesions, and 52.6% of the HCCs were considered widely invasive. Vascular invasion was present in 76% and extrathyroidal extension in 6.5% of the HCCs. Lymph node metastases were diagnosed in 5 cases (10%), while 6 patients (12%) had distant metastases.

**Figure 2:**
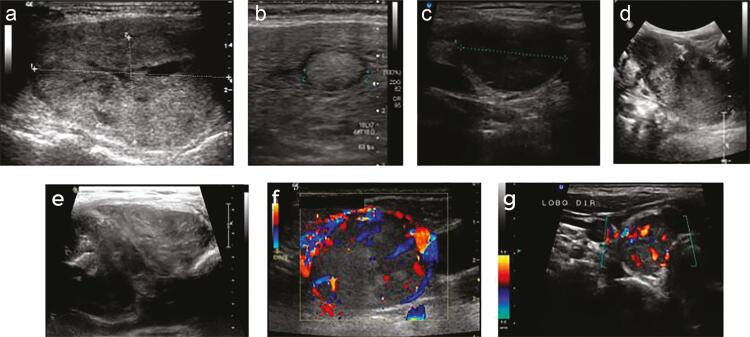
Variety of sonographic presentations of Hürthle cell carcinomas. (A) Partially cystic isoechoic nodule; (B) solid isoechoic nodule with peripheral halo; (C) markedly solid hypoechoic nodule; (D) solid hypoechoic nodule with coarse calcifications; (E) solid hypoechoic nodule with microcalcifications and coarse calcifications; (F) solid isoechoic nodule with peripheral vascularization; (G) solid hypoechoic nodule with intranodular vascularization.

The mean largest dimensions of the carcinomas and adenomas on the ultrasound images were 50.1 ± 28.3 vs. 37.9 ± 22.5 mm (p = 0.05), respectively. The ultrasound features of HCCs and HCAs are described in Table 1; no significant differences were observed between the groups. A wide spectrum of sonographic patterns was observed in carcinomas (Figure 2). A cytological diagnosis of follicular neoplasm/HCN (Bethesda IV) was observed in 87.2% of HCC and 96.7% of HCA cases (Table 2). The diagnostic performance of ultrasound in HCC is presented in Table 3.

**Table 1:** Ultrasound characteristics of Hürthle cell carcinomas and adenomas

Ultrasound characteristic	Carcinoma (n = 24)	Adenoma (n = 27)
Hypoechogenicity (%)	9 (37.5)	6 (22.2)
Purely solid (%)	13 (54.2)	12 (44.4)
Irregular contours (%)	1 (4.2)	3 (11.1)
Absence of hypoechoic halo (%)	5 (20.8)	11 (40.7)
Microcalcification (%)	2 (8.3)	3 (11.1)
Coarse calcification (%)	3 (12.5)	2 (7.4)
Taller-than-wide shape (%)	4 (16.7)	8 (29.6)
Central or predominantly central blood flow (%)*		
TI-RADS	10 (52.6)	12 (60)
2	5 (20.8)	4 (14.8)
3	5 (20.8)	10 (37)
4	12 (50)	10 (37)
5	2 (8.3)	3 (11.1)

* Percentage from available exams.

**Table 2:** Thyroid cytopathology of Hürthle cell carcinomas and adenomas

Cytopathology	Carcinomas (n = 38)	Adenomas (n = 30)	p value
Benign	4 (10.5%)	1 (3.3%)	0.156
Follicular neoplasm or suspicious for follicular neoplasm	9 (23.7%)	12 (40%)	0.231
Follicular neoplasm or suspicious for follicular neoplasm, Hürthle cell	24 (63.1%)	17 (56.6%)	0.587
Nondiagnostic or unsatisfactory	1 (2.7%)	-	-

**Table 3:** Performance of sonographic features raising suspicion for malignancy in the diagnosis of Hürthle cell carcinomas

Ultrasound characteristic	Sensitivity (95% CI)	Specificity (95% CI)	Positive LR (95% CI)	Negative LR (95% CI)	Accuracy (95% CI)
Hypoechogenicity	37.5% (18.80-59.41)	77.8% (57.74-91.38)	1.69 (0.70-4.05)	0.8 (0.56-1.16)	58.8% (44.17-72.42)
Purely solid	54.2% (32.82-74.45)	55.6% (35.33-74.52)	1.22 (0.70-2.13)	0.83 (0.48-1.43)	54.9% (40.34-68.87)
Irregular contours	4.2% (0.11-21.12)	88.9% (70.84-97.65)	0.37 (0.04-3.37)	1.08 (0.92-1.26)	49% (34.75-63.40)
Absence of peripheral halo	20.8% (7.13-42.15)	59.3% (38.80-77.61)	0.51 (0.21-1.26)	1.34 (0.92-1.94)	41.2% (27.58-55.83)
Microcalcification	8.3% (1.03-27.00)	88.9% (70.84-97.65)	0.75 (0.14-4.12)	1.03 (0.86-1.23)	51% (36.60-65.25)
Coarse calcification	12.5% (2.66-32.36)	92.6% (75.71-99.09)	1.69 (0.31-9.26)	0.94 (0.79-1.14)	54.9% (40.34-68.87)
Taller-than-wide shape	16.7% (4.74-37.38)	70.4% (49.82-86.25)	0.56 (0.19-1.63)	1.18 (0.87-1.60)	45.1% (31.13- 59.66)
Central or predominantly central blood flow	50% (27.20-72.80)	36.8% (16.29- 61.64)	0.79 (0.45-1.38)	1.36 (0.65-2.83)	43.6% (27.81-60.38)
TI-RADS 4 or 5	58.3% (36.64-77.89)	51.9% (31.95-71.33)	1.21 (0.72-2.03)	0.8 (0.44-1.46)	54.9% (40.34-68.87)

LR: likelihood ratio; 95 % CI: 95% confidence interval.

Hypoechogenicity and presence of coarse calcifications had the highest positive LR. However, the specificity of hypoechogenicity was only 77.8%. Also, despite having high specificity, the presence of coarse calcifications was observed in only 12.5% of the HCCs.

We also evaluated the diagnostic performance of the TI-RADS classification in HCCs. Even though 58% of the HCCs were classified as TI-RADS 4 or 5, no significant difference was observed in this regard when HCCs were compared with HCAs, as 48% of the latter cases were also moderately or highly suspicious. Therefore, TI-RADS 4 or 5 had a specificity of only 51.8% and a positive LR of 1.21.

Apart from tumor size, no significant difference occurred between benign and malignant cases that could allow an accurate preoperative diagnosis. In this series, Hürthle cell tumors larger than 4 cm had a significantly increased malignancy risk, which reinforces data from a recent report of 330 pathologically diagnosed oxyphilic cell neoplasms (61 carcinomas), in which a tumor size > 4 cm was an independent predictor of HCC (23). Other studies have also demonstrated a correlation between tumor size and malignancy risk in HCNs. A tumor size ≥ 2.5 cm was an independent predictor of HCC (24), whereas all tumors ≤ 2 cm observed were benign and all of those larger than 6 cm were malignant (25).

As previously demonstrated, the cytologic analysis of our tumors failed to identify malignant nodules correctly, as 87% of the HCCs and 97% of the HCAs were diagnosed as follicular neoplasm/HCN (Bethesda IV). The positive predictive value for malignancy of cytologic HCN diagnoses has been previously described to be between 15 and 45% (13,15), decreasing to 9.5% in the presence of Hashimoto’s thyroiditis (14). Therefore, other preoperative aspects should be evaluated to allow a proper diagnosis of malignancy before surgery.

Several sonographic features are usually associated with a higher risk of malignancy in a thyroid nodule. According to the American Thyroid Association, a highly suspicious sonographic pattern is represented by a solid hypoechoic nodule or solid hypoechoic component of a partially cystic nodule associated with at least one of the following findings: irregular margins, microcalciﬁcations, taller-than-wide shape, rim calciﬁcations with a small protruding soft tissue component or evidence of extrathyroidal extension. The absence of a peripheral halo and increased intranodular vascularization are also considered suspicious for malignancy (1). These suspicious characteristics are mainly observed in papillary thyroid carcinomas (1,26).

In this retrospective analysis, B-mode and color Doppler sonographic features were unable to differentiate malignant from benign HCNs. HCNs may present a wide variety of sonographic findings (16-20,24,27), and hypoechogenicity predicted malignancy in this background (28). Ito and cols. (23) observed that round and hypoechoic solid nodules and solid nodules with irregular borders or with psammoma calcifications represented 83% of the carcinomas and 78% of the adenomas. However, the authors concluded that such sonographic presentations were independent predictors of malignancy in patients with Hürthle cells in FNA cytology. In contrast, lesions with a flat, isoechoic, or hyperechoic pattern with a peripheral halo were frequent among the HCCs in the present study at rates of 83.3%, 62.5%, and 79.2%, respectively.

The sonographic features of follicular thyroid carcinomas are different from those of papillary carcinomas. The most frequent presentation of follicular carcinomas (with respective presentation rates for each feature) is as a solid (82.6%), flat (72.7%), isoechoic (65.2%) nodule with a hypoechoic rim (86.6%) and without calcifications (82.6%) (26). The peripheral halo has been suggested to represent the tumor’s capsule or pseudocapsule of surrounding fibrous connective tissue, which could be present in both benign and malignant HCNs (19). Unlike previous data on follicular carcinomas (26), cystic changes (45.8%) and calcifications, mainly coarse ones (12.5%), were observed in our HCCs. Lee and cols. (19) reported cystic changes in around 50% and coarse calcifications in 20% of HCNs, without significant differences between benign and malignant nodules, although the authors analyzed a smaller sample of only 3 HCC. Regarding vascularization, our study showed predominantly intranodular vascularization in both HCCs (52.6%) and HCAs (60%).

We evaluated the performance of the TI-RADS classification in diagnosing HCCs (22). TI-RADS 4 (moderately suspicious) or TI-RADS 5 (highly suspicious) nodules corresponded to 58.3% of our HCCs. However, 48% of the HCAs were also categorized as moderately or highly suspicious according to this classification. Likewise, the Korean TIRADS was also unable to distinguish HCCs from HCAs (29).

This study has some limitations. The retrospective analysis was restricted to pathologically proven HCNs, instead of considering all nodules suspected of HCNs on cytology. Although this series included a higher number of HCCs than other reports (due to the rarity of these tumors), the number of cases could be considered small to identify statistically significant differences.

In conclusion, apart from tumor size, no other preoperative feature was able to differentiate HCCs from HCAs adequately. Sonographic characteristics considered suspicious for malignancy, which are mostly seen in papillary thyroid carcinomas, were infrequent in HCCs. Besides, the combination of suspicious sonographic characteristics with the TI-RADS classification was also insufficient to diagnose the cases of HCC accurately. Therefore, new tools must be developed, such as molecular tests or other imaging tools like elastography or contrast-enhanced ultrasound, to allow an accurate preoperative diagnosis of malignancy and possibly defer diagnostic surgery in cases of adenomas.
